# The Prevalence of Norovirus in returning international travelers with diarrhea

**DOI:** 10.1186/1471-2334-10-131

**Published:** 2010-05-25

**Authors:** Nadja Apelt, Christine Hartberger, Hartmut Campe, Thomas Löscher

**Affiliations:** 1 Department of Infectious Diseases and Tropical Medicine, Ludwig-Maximilians University of Munich, Georgenstr. 5, 80799 Munich, Germany; 2 State Department for Health and Food Safety - Landesamt für Gesundheitsschutz und Lebensmittelsicherheit, Oberbayern, Veterinärstr. 2, Oberschleißheim, Germany

## Abstract

**Background:**

There is a high incidence of diarrhea in traveling populations. Norovirus (NV) infection is a common cause of diarrhea and is associated with 7% of all diarrhea related deaths in the US. However, data on the overall prevalence of NV infection in traveling populations is limited. Furthermore, the prevalence of NV amongst travelers returning to Europe has not been reported. This study determined the prevalence of NV among international travelers returning to Germany from over 50 destinations in and outside Europe.

**Methods:**

Stool samples of a total of 104 patients with a recent (< 14days) history of international travel (55 male, mean age 37 yrs.) were tested for the presence of NV genogroup (GG) I and II infection using a sensitive and well established quantitative RT PCR method. 57 patients experienced diarrhea at the time of presentation at the Department of Infectious Diseases & Tropical Medicine. The remaining 47 patients had no experience of diarrhea or other gastrointestinal symptoms for at least 14 days prior to their date of presentation at our institute.

**Results:**

In our cohort, NV infection was detected in 15.7% of returning travelers with diarrhea. The closer to the date of return symptoms appeared, the higher the incidence of NV, ranging as high as 21.2% within the first four days after return.

**Conclusions:**

In our cohort, NV infection was shown to be frequent among returning travelers especially in those with diarrhea, with over 1/5 of diarrhea patients tested positive for NV within the first four days after their return to Germany. Due to this prevalence, routine testing for NV infection and hygienic precautions may be warranted in this group. This is especially applicable to patients at an increased risk of spreading the disease, such as healthcare workers, teachers or food-handlers.

## Background

An increased incidence of diarrhea can be detected in traveling populations [1]. In industrialized countries noroviruses (NV) are presently recognized as the most common cause of diarrhea in the adult population, both for epidemic [2] and sporadic [3] cases of diarrhea. It is estimated that they cause up to 85% of all viral enteritis worldwide [4]. Despite the high rate of recovery generally associated with NV infection, up to 7% of all diarrhea-related deaths in the US are thought to be caused by NV [5]. Thus, NV enteritis can be expected to cause significant human and economic loss [6,7] which could be exacerbated in patients suffering co-morbid conditions.

Judging by two studies carried out in 2002 and in 2008 the economic and human impact that NV spread by travelers has on European public health systems appears to be substantial[8,9]. As with any rapidly mutating viral agent where pandemics due to newly emerging genotypes are reported nearly every two years, travelers are expected to significantly contribute to the spread of NV. A number of infected pilgrims returning from Lourdes was shown to cause more than 380 secondary cases including one fatality in 29 Swiss nursery homes [8]. In addition, 130 secondary cases, nine outbreaks and four deaths across France, the Netherlands and Ireland were directly attributable to epidemic enteritis spread by returning pilgrims from Lourdes [9].

RT-PCR methods have been recognized as the gold standard of NV detection [10-12]. A report of 35 patients with diarrhea that traveled from the US to Mexico in 2005 showed the exceptionally high rate of NV infection of 65% using a sensitive RT-PCR method [13]. In contrast, previous studies reported infection rates of only 5-15% in travelers suffering diarrhea in the 1980's using radioimmunoassay methods [14-16]. In spite of the discrepancies in the prevalence of NV infection reported amongst travelers, the data suggests that travelers are a particular group that is susceptible to NV infection. This warrants further investigation, especially since, to the best of our knowledge, no data has been published assessing the prevalence of NV amongst European traveling populations. By providing data on the scale of the problem at hand, the study of prevalence rates of NV infection among international travelers returning to Europe will significantly contribute to assessing the impact NV infected travelers have on European public health systems.

## Methods

Fresh stool samples from 104 recently (< 14days) returned travelers with and without diarrhea were collected from August 2006 through May 2008 at the Department of Infectious Diseases and Tropical Medicine in Munich, Germany.

Sample preservation was performed as previously reported for NV preservation from stool [17-21], creating a 20% suspension of the sample in nuclease-free water and storing samples at -80°C until testing. RNA extraction was carried out on the same day of RT-PCR testing. In short, the stool suspension was diluted to 10% using nuclease-free water and centrifuged at 3500 g for 10 minutes. RNA extraction was carried out using the „MicrolabStar“ robot by Hamilton (Bonaduz, Switzerland) and the QiAmp biorobot kit by Qiagen (Hilden, Germany), following the manufacturers manual.

Samples were tested for NV genogroups I and II following a standard qRT-PCR protocol at the 'State Department for Health and Food Safety' as previously published [18]. Qiagen One Step RT-PCR kit was used, following the manufacturers protocols. Primers were synthesized by MWG Biotech (Ebersberg, Germany). Primers NV 192, NV 193 and probe TM8 were used for the detection of genogroup (GG) I viruses and primers 107a, NV 117 (corresponding to NV 119 in [18]) and probe TM3 for the detection of GGII (tab. 1). Reference dye of the reaction was "ROX" (Invitrogen, Karlsruhe, Germany).

**Table 1:** Primer sequences. Primer sequences used for the detection of norovirus. Primers for both genotypes target the ORF1/ORF2 overlap of the viral genome.

Genogroup	Primer	Sequence	Location*
**G I**	NV 192	5'- gC (CT) Atg TTC CgC Tgg Atg C - 3'	5282 - 5300
**G I**	NV 193	5'- CgT CCT Tag ACg CCA TCA TCA - 3'	5379 - 5359
**G I**	TM8	5'- FAM - Tgg ACA gg(Ag) gAT CgC (Ag)AT	5321 - 5345
	Probe	CTC CTg C - 3' - TMR	
**G II**	NV 107a	5'- AgC CAA TgT TCA gAT ggA Tg - 3'	5007 - 5026
**G II**	NV 117	5'- TCg Acg CCA TCT TCA TTC AC-3'	5100 - 5081
G II	TM3	5'- FAM - Tgg gAg ggC gAT CgC AAT CTg gC -	5048 - 5070
	Probe	3' - TMR	

*Genome location of primers and probes are based for GI on the sequence of Norwalk/68/US [GenBank: M87661] and for GII on the sequence of Lordsdale/93/UK [GenBank: X86557];

Ethical clearance for the study was sought through the Ethical Committee of the Medical Faculty at Ludwig-Maximilians University, Munich, Germany. Clinical and laboratory data was used only in the case that patients had provided written informed consent to participate in this study or after written informed consent was obtained from the legal caretakers in the case of minors.

Statistical evaluation was carried out using SPSS 13.0 for Windows "student version" as well as standard applications of Microsoft Excel Version 2003.

## Results

Of the 104 patients included in this study 55 were male. Sex was evenly distributed between patients with and without diarrhea (p = 0.652; chi-square test). The study included patients from 4 to 80 years of age (mean 37.8 ± 14.7 yrs) without significant differences between patients with and without diarrhea (p = 0.473; one-way Anova test). The mean duration of the journey was comparable in patients with and without diarrhea (p = 0.949 one-way Anova test) with 69 and 71 days, respectively. (tab. 2)

**Table 2:** Overview of results. Demographic data and frequency of norovirus (NV) infection in recently returned travelers with diarrhea and in a control group of recently returned travelers without diarrhea:

	Patients with diarrhea (n = 57)	Asymptomatic patients (n = 47)	Differences between groups
**Male**	29	26	p = 0.652
**Mean age (yrs)**	38.7 ± 15.7	36.6 ± 13.4	p = 0.437
**Mean duration of journey (days)**	68.5 ± 166	71.2 ± 266	p = 0.949
**NV infection**	9 (15.7%)	1 (2.1%)	**p = 0.019**
**GGI**	1	0	
**GGII**	7	1	
**GGI + GGII coinfection**	1	0	
**Traveling modality and corresponding odds of infection**	Backpacking trip: OR = 4.923 Business trip: OR = 1.429 Packaged holiday: OR = 0.304		
**Time until presentation at clinic after return (in days)**	4.7 ± 3.0	5.3 ± 3.5	p = 0.330
**India (total/NV+)**	13/4	8/1	
**Backpacker (total/NV+)**	22/6	6/0	

Overall 10 cases of NV infection were detected in this study, nine of which were found in patients with diarrhea (15.7%), and one in the group of patients without diarrhea (2.1%). Of the 10 cases of NV detected, eight were caused by GGII viruses alone, one was a coinfection with GGI and GGII and one case was an infection caused by GGI alone. As expected, the difference in the number of NV infections proved significant when comparing patients with and without diarrhea (p = 0.019 by chi-square test). Both sexes were equally likely to be infected (p = 0.210, chi-square test). (tab. 2)

Of the nine positively tested patients that suffered diarrhea the following symptoms in addition to diarrhea were reported to the observing physician: fever (2), abdominal cramping (2), nausea (4), vomiting (2), headache (2) and myalgia (2).

Patients in this study visited a total of over 50 destinations. Traveling modality was shown to influence the odds of infection. All patients with diarrhea and 20 patients without diarrhea provided information as to their traveling modalities. Overall, 28 patients (36%) had taken part in a back-packing/adventure trip, 19 (25%) had participated in a packaged holiday, 13 (17%) had been on a business trip and eight (10%) were visiting friends and family. All other patients that gave information as to their way of traveling had either traveled as missionaries or exchange students. Comparing traveling modalities within the group of recently returned travelers with diarrhea, the backpacking trip was associated with the highest risk of a positive NV test result, when compared to other forms of traveling (OR = 4.923). The business trip carried the second highest risk of infection (OR = 1.429), and those who had traveled on a packaged holiday carried the lowest risk of a positive test result (OR = 0.304). (tab. 2)

**Figure 1:**
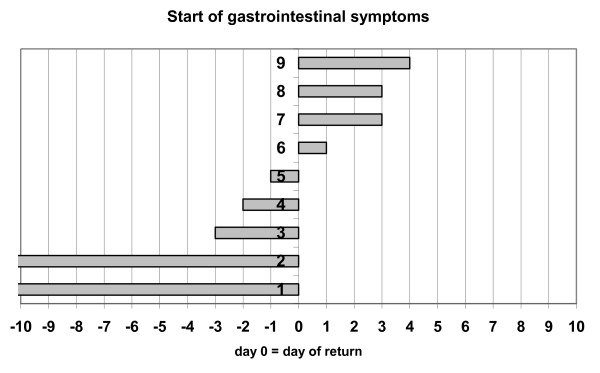
**Symptom duration in relation to the date of return**. Graphic representation of the start date of gastrointestinal symptoms for all nine returning travelers with diarrhea. Patients enlisted as Patients 1 and 2 in this graph have claimed to have suffered from gastrointestinal symptoms for more than ten days before their return to Germany.

It took patients with diarrhea 4.7 ± 3.0 days (minimum 0, maximum 13; 95% confidence interval 3.92-5.52 days) and patients without diarrhea 5.3 ± 3.5 days (min. 0, max. 14; 95% confidence interval 4.32-6.36 days) to present at our clinic for the first time after returning to Germany. These time spans are comparable for both groups (p = 0.330 One-way Anova test). (tab. 2) Eight of the nine positively tested patients with diarrhea had visited the clinic within the first five days after their return. Five of nine diarrhea patients which were tested positive claimed to have suffered from diarrhea since before their return to Germany, the remaining four declared to have started experiencing symptoms within the first four days after their return. (Fig. 1) 21.2% of diarrhea patients tested for NV within the first four days after their return to Germany tested positive. There was no significant correlation between the duration of the journey and the likelihood of NV infection.

## Discussion

NV enteritis is generally recognized as an acute self-limiting disease inducing vomiting and watery non-bloody diarrhea lasting 12 to 60 hours. Recovery is usually complete and complications are rare in the non-comorbid adult population [12,22-25]. However, it has been shown that infants, elderly and immunocompromised patients, and those suffering cardiovascular disease are significantly more likely to experience a complicated or fatal course [12,26]. The most common consequence of the disease is dehydration, even though secondary complications as diverse as aspiration pneumonia, renal failure, mucositis and acute abdomen have been described [12,26].

Pandemics caused by newly emerged genotypes of NV, in particular genotype GII.4, have been reported approximately every two years since 2002 [27]. This is thought to be due to the high mutation potential of NV. Indeed the mutation rate of NV has been quoted to be significantly higher than that of influenza viruses [28]. The present study shows a high rate of NV infection in travelers returning to Germany suffering diarrhea. Overall, 15.7% of diarrhea patients were infected with the virus. As a comparison, only four in 79 travelers (2%) returning from the US to Sweden with respiratory symptoms were shown to suffer from influenza type A (H1N1) in a recent survey [29].

Our results support previous findings of a high prevalence of NV among US travelers for the first time in a European population and compare to findings quoted in a recent review [30]. Furthermore we show that in this population, the closer to the date of return diarrhea developed, the higher the rate of NV+ cases, reaching 21% for patients suffering diarrhea within four days after their return.

NV is a common cause of diarrhea in the adult population. Thus, when evaluating the frequency of NV in a traveling population it should always be kept in mind that NV may be equally prevalent in a non-traveling population. In an unpublished in-house study at the Department of Infectious Diseases and Tropical Medicine the prevalence of NV unrelated to travel was assessed in 48 diarrhea patients using the method described in this paper (personal communication). As the largest part of patients at our clinic possesses a clinical history of recent travel, a cut-off point was established to define patients whose symptomatic NV infection was unlikely to be linked to travel. This cut-off point was chosen at 14 days after return from the last international journey and NV infections in patients that presented to the clinic 14 days or more after their return were considered to be unrelated to travel. This choice was based on the maximum incubation time reported for NV that was estimated at 130 hours [31] and the mean duration of symptoms for the common GII.4 virus group that was reported to average 69 hours [32]. In this group of 48 diarrhea patients a single patient has been found to suffer NV infection (2.1%). The low prevalence in this cohort may indicate that traveling might be an independent risk factor for the contraction of NV.

It was a special interest of ours to identify those diarrhea patients at highest risk for NV among the inhomogeneous group of returning travelers. The Department of Infectious Diseases and Tropical Medicine routinely collects data on the mode of traveling of patients. Hence, we could observe participants in backpacking/adventure trips to be the most likely to have contracted NV infection when compared to other returning travelers with diarrhea (OR = 4.923). Having traveled on a packaged holiday lowered the likelihood of NV when experiencing diarrhea (OR = 0.304). We also consider it remarkable for 30.1% of returnees from India suffering diarrhea to have been tested NV+, even though we are fully aware of the fact that the scale of this investigation does not allow any conclusions as to the likelihood with which NV affects visitors to specific destinations.

## Conclusion

A prevalence of NV of 21.2% among diarrhea patients within the first four days after their return from an international journey may be sufficiently high to warrant routine testing for NV in this group. Recent studies suggest that NV is continuously shed for an average of 28 days after symptoms subside [31], even though detection techniques may oftentimes not be sensitive enough to detect viral shedding for more than 14 days after the end of symptoms. However, bearing in mind that 10-100 viral particles are sufficient to induce symptomatic NV infection [33,34] it is highly likely that patients remain infectious for all of the 28 days that virus may be shed. Thus routine NV testing in populations where NV is highly prevalent may serve as a measure of public health prevention. It allows doctors to take preventive action in the case of a positive test result, especially were persons at increased risk for spreading the disease such as health care workers, teachers and food handlers are concerned.

## Competing interests

The authors declare no existing conflict of interests. No third party funding has been used in the generation of the presented data.

## Authors' contributions

All authors have read and approved of the final manuscript and have contributed to the presented work as follows. NA carried out the sample collection, sample testing, statistical analysis and drafted the manuscript. NA also participated in study design. CH carried out the sample testing while HC provided machines, reagents and facilities for sample testing. TL conceived of the study and participated in its design and coordination. TL also contributed to the drafting of the manuscript.

## Pre-publication history

The pre-publication history for this paper can be accessed here:

http://www.biomedcentral.com/1471-2334/10/131/prepub
